# Role of Solvent Used in Development of Graphene Oxide Coating on AZ31B Magnesium Alloy: Corrosion Behavior and Biocompatibility Analysis

**DOI:** 10.3390/nano12213745

**Published:** 2022-10-25

**Authors:** Muhammad Faheem Maqsood, Mohsin Ali Raza, Zaeem Ur Rehman, Asima Tayyeb, Muhammad Atif Makhdoom, Faisal Ghafoor, Umar Latif, Muhammad Farooq Khan

**Affiliations:** 1 Institute of Metallurgy & Materials Engineering, Faculty of Chemical & Materials Engineering, University of the Punjab, Lahore 54590, Pakistan; 2 Faculty of Nanotechnology and Advanced Materials Engineering, Sejong University, Seoul 05006, Korea; 3 Department of Electrical Engineering, Sejong University, 209- Neungdong-ro, Gwangjin-gu, Seoul 05006, Korea; 4 School of Biological Sciences, Quaid-e-Azam Campus, University of the Punjab, Lahore 54590, Pakistan

**Keywords:** magnesium alloys, graphene oxide, atomic force microscopy, electrochemical characterization, cell viability, biocompatibility

## Abstract

Clinical applications of bio-absorbable magnesium (Mg) and its alloys can be enhanced by increasing their corrosion resistance, using surface modification and functionality. In this study, we synthesized graphene oxide (GO) through improved Hummers’ method and deposited it on biodegradable AZ31B Mg alloy for further characterization. Different suspensions of GO were prepared in various solvents, like deionized water, ethanol, and acetone by ultra-sonication. Electrophoretic deposition (EPD) was used to develop GO coatings on AZ31B Mg using different GO suspensions. Effect of various solvents on corrosion behavior, as well as in vitro biocompatibility, was studied. The optimized EPD parameters were 3 volts and 90 s for coating. Different characterization techniques were used to study GO and prepared coatings. Atomic force microscopy found that the average thickness of GO was ~1 nm. Electrochemical behavior of coatings was studied through electrochemical impedance spectroscopy (EIS) and Tafel analysis in Ringer’s lactate solution. Tafel analysis revealed that GO coatings deposited by GO water suspension increased corrosion protection efficiency of AZ31B Mg alloy by ~94%. After 72 h incubation in MC3T3-E1 osteoblast cells extract, in vitro analysis was performed to determine the cell viability and biocompatibility of the GO- coated and bare Mg samples. GO coatings deposited by GO water suspension demonstrated ~2× cell viability, as well as nontoxicity and better biocompatibility compared to the bare and other GO-coated Mg samples.

## 1. Introduction

Magnesuim (Mg) and its alloys bearing high specific strength and good biodegradeability are the widely explored green engineering materials [1,2,3]. Mg alloys exhibit low corrosion and wear and tear resistance [4,5]. The biodegradeability in Mg alloys is manifested as a result of the presence of instinctive oxide and OH^-^ film on the surface of Mg alloy which is highly unreliable and cannot remain intact in human bodily fluids to preserve Mg alloy [6]. Human bodily fluids contain active ions (Cl^−^ and NO_3_^−^) and the Mg alloy has higher negative self-corrosion potential, which makes it a anode (in galvanic cell system) and results in the rapid dissolution of Mg alloy [7,8]. The faster disbanding of Mg alloy may cause many tribulations, e.g., glut of hydrogen evolution near cavities of the wounded portion make bubbles after implantation, and these bubbles can deteriorate the performance of the implant [9] despite disappearing after a few weeks [10,11]. Likewise, local alkalization around the surface of Mg-based implants, due to high hemolysis of red blood cells, [12] may be fatal for living organisms, as pH grows in relation to concentrated alkaline micro-environments [9]. Furthermore, the mechanical strength of the Mg alloy decreases with increasing [Mg^2+^] as a result of the elevation in the osmotic pressure of the human bodily fluid, thus causing low cell viability [12,13] and structural imperfection of Mg alloys [14] which results in mechanical pre-failure of the implant [15]. 

Properties like biodegradation, biocompatibility, bioactivity, and adaptation of Mg alloys can be standardized up to an estimated level by adjusting the corrosion rate with the aid of suitable coating such as hydroxyapatite, epoxy silane, graphene, etc. [16,17,18,19]. Graphene, a two-dimensional (2D) material, exhibits a honeycomb structure made up of a single layer of carbon atoms. It has been extensively explored owing to its bio-stability, non-toxicity, massive external surface area, impressive mechanical properties, and anti-corrosion properties [20,21,22,23,24,25]. Owing to functional groups such as hydroxyl, epoxide, carboxyl and carbonyl, graphene derivatives such as graphene oxide (GO) have better capability to disperse in water to form steady suspension [26], while the coating of these materials can be crafted by electrophoretic deposition (EPD), chemical vapor deposition (CVD), anodic oxidation, and physical vapor deposition (PVD) [27,28,29,30,31,32]. 

For surface treatment of magnesium alloys, selection of environmentally friendly components becomes one of the focuses of research [33]. EPD is a practical, environmentally friendly and effortless technique to fabricate GO coatings on metallic substrates for corrosion protection application [34,35,36]. In recent years, many researchers have devoted their attention to GO owing to its easy aqueous dispersion formation, high chemical activity, and excellent lubrication property [37,38,39]. An et al. [36] studied the corrosion resistance of GO coating on stainless steel developed by EPD. Raza et al. [40] demonstrated that the GO-EPD coating on copper slashed corrosion rate by 6× in relation to plain Cu in 3.5% NaCl. Rather than corrosion properties, coatings of graphene and their derivatives play a role in cell viability, metabolic activity, toxicity, and biocompatibility of underlying material. It has already been established that the GO does not cause any harmful effects like cytotoxicity or genotoxicity to different cells [41]. Li et al. [42] used cathodic EPD to fabricate GO/hydroxyapatite (HA) coatings on pure titanium (Ti) substrate. They observed that the GO in HA coating reduced surface cracks, improved coating adhesion, and provided better corrosion protection in simulated bodily fluid. Moreover, they found that 2 wt.% GO/HA films gave superior in vitro biocompatibility (around 95% cell viability of L929 and MG63 cells) compared to other compositions, e.g., neat HA coating and Ti substrate. Carpio et al. [43] studied the antimicrobial activity of silanized GO with N (trimethoxysilylpropyl) ethylenediamine triacetic acid against both types of bacteria, as well as its cytotoxicity to human corneal epithelial cell line (hTCEpi) and found improved anti-microbial properties with no cytotoxicity, even after 24 h exposure. Ordikhani et al. [44] reported that the GO/chitosan nanocomposite coatings deposited on Ti by EPD demonstrated high biocompatibility compared to neat chitosan coating and bare Ti film. 

This research aims to produce excellent biodegradable graphene oxide (GO)-coated magnesium (Mg) AZ31B alloy with good corrosion resistance and suitable biocompatibility. Effect of different solvents (deionized water, ethanol, and acetone) on the development of GO coatings and their corrosion performance and biocompatibility of coated Mg alloy was studied. The main objective of this research is to decrease the degradation rate of Mg alloy without any harmful side effect for body implants. Further, standard 3-(4,5-dimethylthiazol-2-yl)-2,5-diphenyl tetrazolium bromide (MTT) assay protocol was used to analyze cell viability, proliferation, and cytotoxicity of bare and GO-coated Mg samples. 

## 2. Experimental

### 2.1. Materials and Reagents

Graphite powder of 10 μm particle size (Asbury Graphite Mills, Old Main St, Asbury, NJ, USA) was used as a precursor to produce graphite oxide. KMnO_4_ 99.0%, H_2_SO_4_ 98.0%, H_3_PO_4_ 99.0%, acetone 99.9%, ethanol 99.8%, and H_2_O_2_ 30.0 wt.% in H_2_O were purchased from Merck KGaA, Darmstadt, Germany. An improved Hummers’ method was used to prepare graphite oxide, then GO [45,46]. Oxide layers and organic contaminants were removed from the surface of Mg samples through grinding and cleaning. 

Graphite oxide was ultra-sonicated separately in DI water, ethanol, and acetone (2 mg/mL) for 2 h at 35 °C to yield different GO suspensions for coating in EPD cell (Figure S1), Mg samples functioned as anode, while platinum worked as cathode. Both electrodes were coupled with DC power supply and dipped in GO solution by maintaining 2 cm distance. EPD was performed at adjusted parameters reported elsewhere [32], 3 V for 90 s, to acquire distinct GO coating. Henceforth, EPD-GO coated samples with different prepared suspension, acetone, ethanol, and DI water were labelled as EPD-GO-A, EPD-GO-E, and EPD-GO-W, respectively. Mechanism for GO deposition on Mg alloy was already stated in our previous research work [32]. 

### 2.2. Characterization

Diffraction patterns of Graphite oxide, EPD-GO-coated Mg, and bare Mg alloy samples were taken by using X-ray diffraction (XRD) [Equinox 2000, Thermo Fisher Scientific, Waltham, MA, USA]. Roughness and thickness of GO and GO-coated Mg samples were assessed by AFM [Nano-Solver, NT-MDT, Moscow, Russia] operating on tapping mode. For the AFM of GO powder (sheets), the suspension of GO in ethanol was used after its ultra-sonication for 2 h at 35 °C, where the 1 to 2 drops of very dilute suspension were drop casted on mica sheet. All AFM scanning images were examined by employing Nova Px 2.0. software (Moscow, Russia) from NT-MDT. The morphologies of GO and EPD-GO-coated Mg samples were explored by SEM (Inspect S50, FEI, Hillsboro, OR, USA), while energy-dispersive X-ray analysis (EDX) was implemented to verify their chemical compositions. 

Raman analysis was done by using Horiba Jobin (JY) RAM Aramis (Kyoto, Kyoto, Japan) confocal having a wavelength of 514 nm. Electrochemical performance of the coatings was probed in a three-electrode cell system by employing Potentiostat/Galvanostat/ZRA [Reference 3000, Gamry Instruments, Warminster, PA, USA] equipped with Echem Analyst (version 7.2) software, where the working electrodes were bare and GO-coated samples, the counter electrode was a graphite rod, and the reference electrode was saturated calomel soaked in Ringers’ lactate solution. Corrosion rate was uncovered by running Tafel scan tests at a a scan rate of 3 mV/sec in a potential range of −0.5 V to +0.5 V vs. open-circuit potential (OCP). EIS was operated in the frequency span of 10 mHz to 100 kHz, with a potential agitation of ±10 mV.

To identify the cell toxicity/biocompatibility of uncoated and GO-coated Mg samples, the mouse MC3T3-E1 cell line from American Type Culture Collection (ATCC) was used. Prior to the direct cell seeding process on the samples, the samples were sterilized under UV radiation for at least 2 h. 1 × 10^6^ cells per ml were seeded in a 96 well plate and incubated in Dulbecco’s Modified Eagle’s Medium (DMEM, Gibco, Thermo Fisher Scientific, Waltham, MA, USA), accompanied with 10% fetal bovine serum (FBS) and 5% Penicillin/Streptomycin in a humidified atmosphere with 5% CO_2_ at 37 ± 1 °C. Six wells were assigned to each group of samples, while cells cultured with normal DMEM were taken as control (negative control). Cell viability was evaluated by using the standard MTT assay protocol after 72 h incubation. Olympus fluorescence microscope (Tokyo, Japan) was used to observe morphology and attachment of cells. After 72 h, MTT, at a concentration of 10 µL, was added per well and was left to rest to allow crystallization for 4 h and further; upon removal of MTT, these crystals were left to dissolve in 100 µL (CH_3_)_2_SO per well for almost 15 min and were transferred to the ELISA reader plate (BioTek microplate reader, Winooski, VT, USA) for measurement of absorbance of each well at 490 nm wavelength. The percentage of cell viability was determined using the following Equation (1), where *OD* represents the optical densities [47].

(1) $$\mathrm{Cell}\;\mathrm{Viability}\;(\%)=\frac{{OD}_{sample}}{{OD}_{control}}\times 100\%$$

## 3. Results and Discussion

### 3.1. XRD

**Figure 1:**
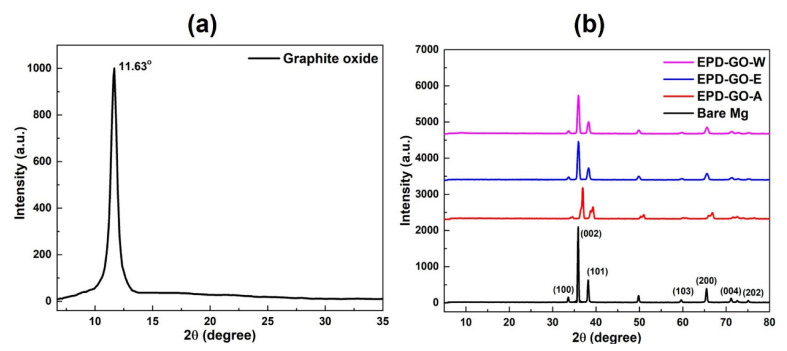
XRD pattern of (**a**) graphite oxide and (**b**) uncoated and EPD-GO-coated Mg samples.

Graphite oxide’s XRD pattern is exhibited in Figure 1a. The diffraction peak at 11.63° relates to (001) plane of graphite oxide by maintaining a d-spacing of 0.77 nm, in contrast to d-spacing of 0.32 nm for natural graphite with (002) plane [48]. Modification in crystal structure and d-spacing occurred due to the heterogeneous nature of graphite oxide. Oxidized graphite with augmented d-spacing was extended to yield GO nanosheets through exfoliation, which was instigated by ultra-sonication in suitable aqueous solvents [40,49]. Figure 1b demonstrate XRD patterns for uncoated and EPD-GO-coated Mg samples. (002) and (001) diffraction peaks were absent in acquired patterns of graphite or graphite oxide, a phenomenon which may be attributed to the minuscule content of GO in coatings to be observed [50,51,52]. It has already been studied previously that the integration of GO into the coating does not ensure the establishment of a new phase [32,53,54,55]. Here, bare and EPD-GO-coated Mg samples only indicated presence of α-Mg peaks matching AZ31B Mg alloy. However, we assume that the reduction in intensities of peaks, shifting of peaks, and broadening of peaks for EPD-GO-coated samples suggests that the deposition of GO was turbstratic (partially crystalline) and had layers arranged coarsely parallel to each other, especially in the case of EPD-GO-A. This agreed with AFM and SEM results, but there is also some arbitrary rotation and translation about the layer. Raman spectra of synthesized GO and EPD-GO-W sample are presented in Figure S2, which confirmed mess in the structure of graphite due to oxidation and presence of multilayer GO sheets.

### 3.2. AFM

**Figure 2:**
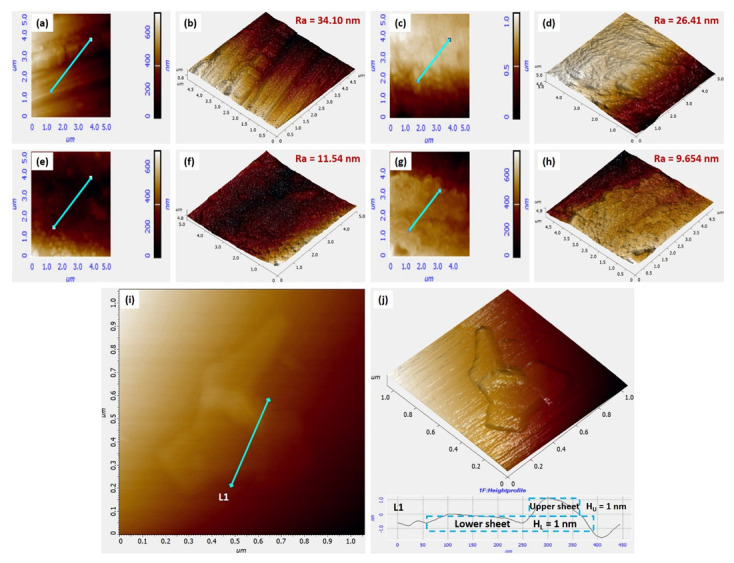
AFM images of (**a**) 2D and (**b**) 3D profile of bare Mg with roughness value, (**c**) 2D and (**d**) 3D profile of EPD-GO-A coated Mg sample with roughness value, (**e**) 2D and (**f**) 3D profile of EPD-GO-E coated Mg sample with roughness value, (**g**) 2D and (**h**) 3D profile of EPD-GO-W coated Mg sample with roughness value, (**i**) 2D & (**j**) 3D profile of GO sheets with height profile.

The morphology of EPD-GO-coated Mg samples and GO sheets was scanned by semicontact AFM mode. Figure 2i,j present the height profile and 3D morphology of GO sheets. GO nanosheets acquired thickness of ~1 nm and a lateral dimension of ~400 nm. Surface roughness, being a vital parameter, was evaluated to estimate coating adhesion and coverage on substrate [56,57]. Figure 2a–h show the morphology and surface roughness values of EPD-GO-coated Mg samples, as well as of the bare Mg sample. Mean roughness (R_a_) of bare Mg, EPD-GO-A, EPD-GO-E, and EPD-GO-W were 34.10 nm, 26.41 nm, 11.54 nm, and 9.65 nm, respectively. R_a_ of EPD-GO-A sample was higher than other coated samples, as sonication of graphite oxide in acetone was not good as it was in ethanol and water. There were chunks and particles of graphite oxide remaining besides GO sheets in acetone suspension. The high R_a_ of EPD-GO-A coating compared to that of other EPD-GO coatings suggests that there was more unsystematic staking of GO layers accomplished during EPD process. However, EPD-GO-W coating was the best, owing to better dispersion of GO sheets in water after quick ultrasonication of graphite oxide. The smoother EPD-GO coatings suggest GO coatings were homogenous and provided the underlying Mg substrate with more coverage as a result of its high surface area [32,58].

**Figure 3:**
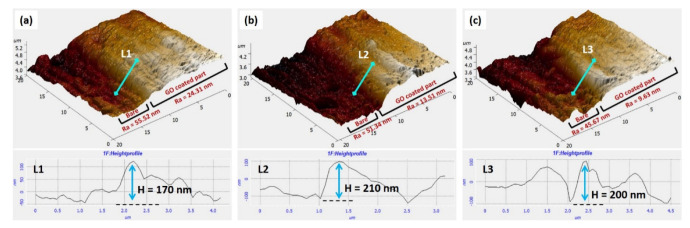
AFM of coating interfaces showing coating thickness along with roughness values for (**a**) EPD-GO-A coating, (**b**) EPD-GO-E coating, and (**c**) EPD-GO-W coating.

Figure 3 shows the thickness and roughness of bare and EPD-GO coated sides of Mg samples. Average thickness of EPD-GO-A, EPD-GO-E, and EPD-GO-W were 170 nm, 200 nm, and 200 nm, respectively. Due to poor ultrasonication of graphite oxide in acetone, there were fewer GO sheets produced, so the thickness of coating was not good as compared to other EPD-GO coatings. R_a_ values of EPD-GO coatings were similar at low and high scan areas (5 μm × 5 μm and 20 μm × 20 μm). 

### 3.3. SEM

**Figure 4:**
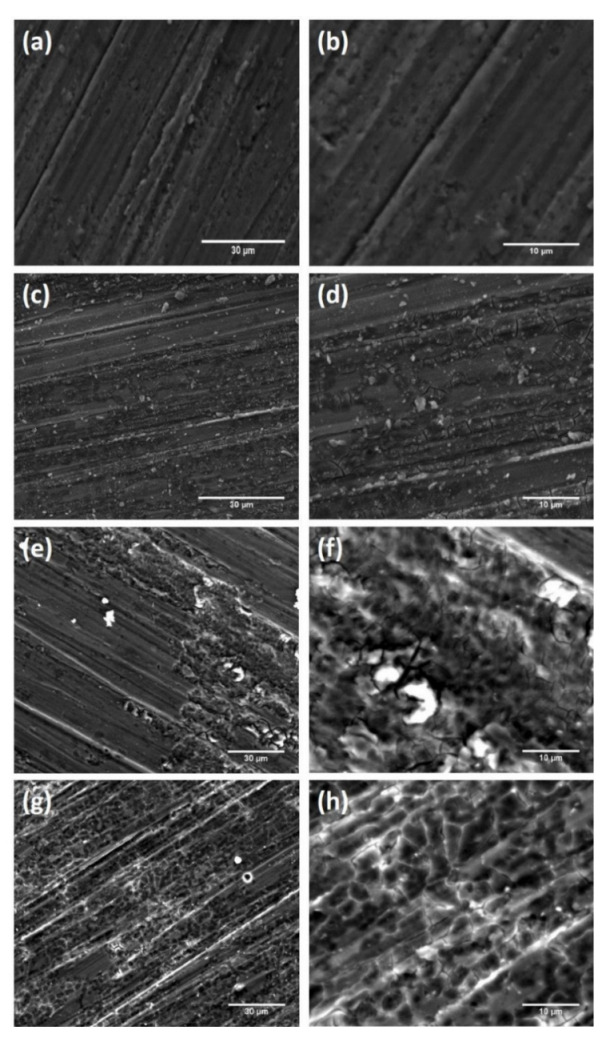
SEM images of (**a**,**b**) uncoated Mg, (**c**,**d**) EPD-GO-A coating, (**e**,**f**) EPD-GO-E coating, and (**g**,**h**) EPD-GO-W coating.

**Figure 5:**
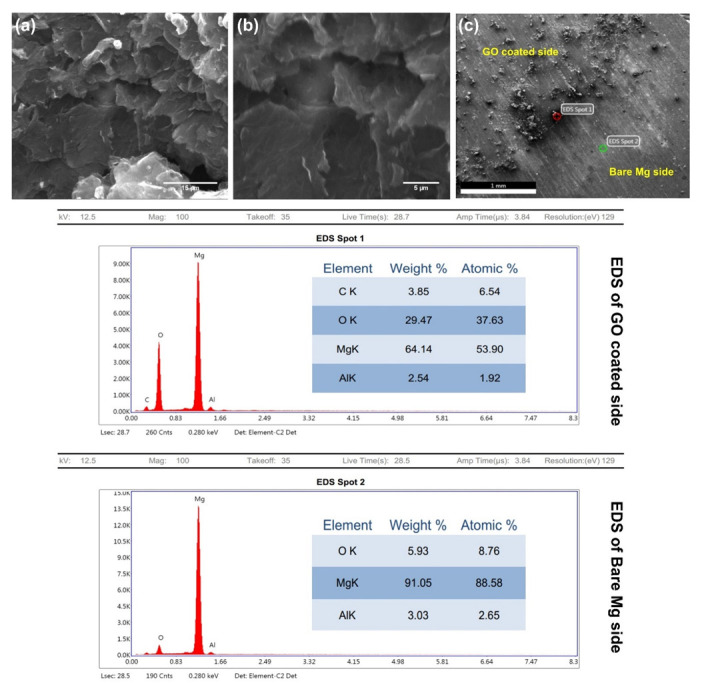
SEM images of (**a**,**b**) GO sheets and (**c**) EDX spectrum of uncoated Mg and EPD-GO-E-coated area.

SEM images of bare Mg and EPD-GO-coated Mg samples are presented in Figure 4. The bare Mg sample exhibits grinding marks, which were developed during sample preparation and resulted in an increase in sample roughness, which, in turn, increased GO coating adherence to Mg substrate (Figure 4a,b). Wrinkles and surface coverage are vital parameters for qualitative investigation of GO coatings [58,59]. Figure 4c,d show SEM images of EPD-GO-A coating on Mg substrate. It is clear from these images that the ultrasonication of graphite oxide in acetone was not good enough, and GO sheets were not properly exfoliated; as a result, coverage of Mg substrate was not achieved properly. These images clearly show improper ultrasonicated graphite oxide particles with coating of GO sheets. Figure 4e–g illustrate SEM images of EPD-GO-E and EPD-GO-W coatings on Mg substrate with better surface coverage. A few graphite oxide particles appear present in the EPD-GO-E coating, distinctly emphasizing that the ultrasonication of graphite oxide in water and ethanol was much better than in acetone. Instead of high grinding marks, in all EPD-GO-coated SEM images, wrinkles and cracks appear, because of the anodic reduction of GO sheets which causes CO_2_ gas evolution at anode as reported by An et al. [36] and Diba et al. [60]. These imperfections might occur due to the nucleation of GO at different sites on Mg substrates to produce various GO domains; subsequently, these domains grow and combine to form GO coating. Due to high surface roughness of the underlying Mg substrate, these domains did not merge impeccably to form smooth GO coating. As a result, cracks were formed [58]. SEM images of GO sheets at high and low magnification are shown in Figure 5a,b. Figure 5c depicts EDX analysis of uncoated and EPD-GO-E-coated areas of Mg substrate. Presence of carbon and high proportion of oxygen at the coated side clearly illustrate that the deposited coating is of GO. 

### 3.4. Electrochemical Testing

#### 3.4.1. Tafel

**Figure 6:**
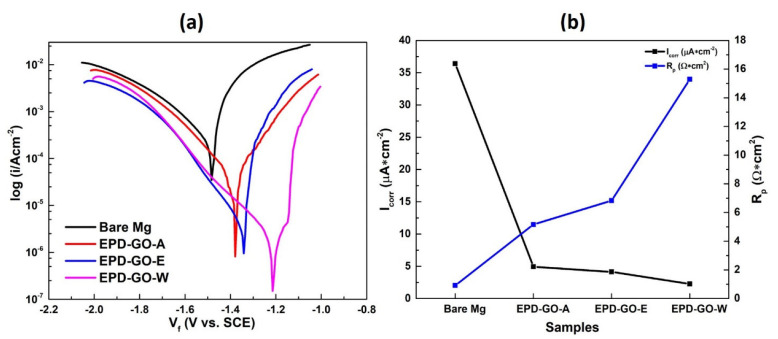
(**a**) Tafel curves and (**b**) I_corr_ and R_p_ values graph of uncoated and EPD-GO-coated Mg samples.

Corrosion rates of bare and EPD-GO-coated Mg samples were appraised using Tafel analysis in Ringer’s lactate solution and are shown in Figure 6a. Table 1 displays kinetic parameters and their standard deviation as determined by Tafel fitting. β_a_, β_c_, I_corr_, E_corr_, and R_p_ represent anodic Tafel slope, cathodic Tafel slope, corrosion current density, corrosion potential, and polarization resistance, respectively. To determine the corrosion rate, Echem Analyst software was employed around the neck of the polarization curves that fit the Butler-Volmer equation [32,61,62]. This region portrays an exponential dependence of current on voltage change in relation to open circuit potential (OCP). Herein, the values of β_a_ and β_c_ are very important to determine whether there are any adsorb species or reaction products at the substrate surface, playing their part in the corrosion rate. β_a_ and β_c_ were obtained from the linear region of anodic and cathodic branches, and show a charge transfer process during their respective polarizations [63]. The value of β_a_ increases if the coating shows some passive performance, while increments in β_c_ value show the coverage of substrate by some reaction products. Hence, it is evinced from Table 1 that the prepared EPD-GO-coated samples will be passive and there will be no reaction products remaining on the coatings, which favor the anodic reaction in the activation polarization region. The value of R_p_ was estimated using Stern-Geary Equation (2) [50,64]

(2) $$R_{P}=\frac{\beta_{a}\times \beta_{c}}{2.303\times \left(\beta_{a}+\beta_{c}\right)\times I_{\mathrm{corr}}}$$

As shown in Figure 6a and Table 1, the bare Mg sample exhibited an E_corr_ value of −1.48 V, and a relating I_corr_ value of 36.40 μA/cm^2^. In contrast to the bare Mg, insignificant increases in the E_corr_ values (positive shift) of EPD-GO-coated Mg samples were observed, which disclose the partial reduction of the thermodynamic tendency of the corrosion emergence [53,54,65]. This positive shift is ascribed to more uniformity and coverage of GO as revealed by AFM and SEM analysis (Figure 2, Figure 3, Figure 4 and Figure 5). Also, the I_corr_ values for the EPD-GO-coated Mg samples were 8 to 16 times lower than those for the bare Mg sample, implying the improvement in the corrosion-resistant properties of the EPD-GO-coated Mg samples. Hence, from Table 1, the corrosion tendency is as follows: bare Mg > EPD-GO-A > EPD-GO-E > EPD-GO-W. In addition, Figure 6b presents the graph between corrosion current density and polarization resistance values, the values of R_p_ seemingly improving from 0.916 Ω×cm^2^ for the bare Mg sample to a range between 5.167 Ω×cm^2^ and 15.29 Ω×cm^2^ for the different EPD-GO-coated Mg samples. This was further vital confirmation for the improved chemical inactivity of the EPD-GO coatings. The decrease in corrosion rate of AZ31B Mg alloy observed here is consistent with prior research [32,66,67]. 

**Figure 7:**
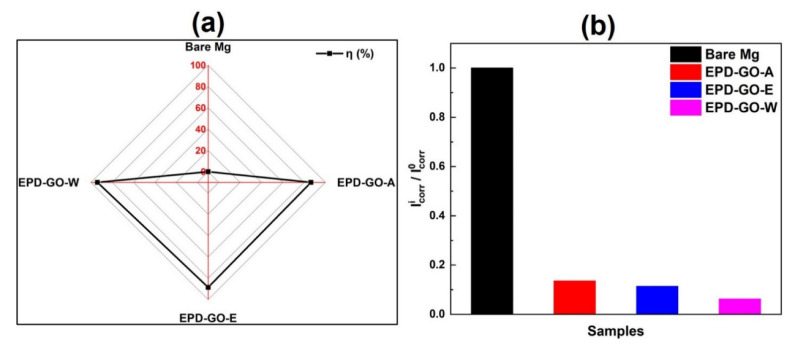
(**a**) Graph of corrosion protection efficiency (η %) as compared with bare Mg and (**b**) current density ratios of the bare and EPD-GO-coated Mg samples.

The corrosion protection efficiency (η %) of a sample depends on the I_corr_ of bare and coated samples, and can be calculated by following Equation (3) [68]:

(3) $$\eta =\left|\frac{I_{\mathrm{corr}}^{i}-{\;I}_{\mathrm{corr}}^{0}}{I_{\mathrm{corr}}^{0}}\right|\times 100\%$$

where $I_{\mathrm{corr}}^{0}$ and $I_{\mathrm{corr}}^{i}$ show current density of uncoated and EPD-GO-coated Mg samples, respectively. EPD-GO-W expresses the best corrosion inhabitation efficiency = 93.79% (see Table 1), and it is validated with avg. corrosion rate and the ratio of EPD-GO-coated and bare Mg sample’s current densities ($I_{\mathrm{corr}}^{i}$/$I_{\mathrm{corr}}^{0}$). The lowest current density ratio = 0.062 for the EPD-GO-W sample and other prepared (0.135 and 0.114 for EPD-GO-A and EPD-GO-E, respectively) coated samples confirms their better anticorrosion property compared to bare Mg (Figure 7).

#### 3.4.2. Electrochemical Impedance Spectroscopy (EIS)

**Figure 8:**
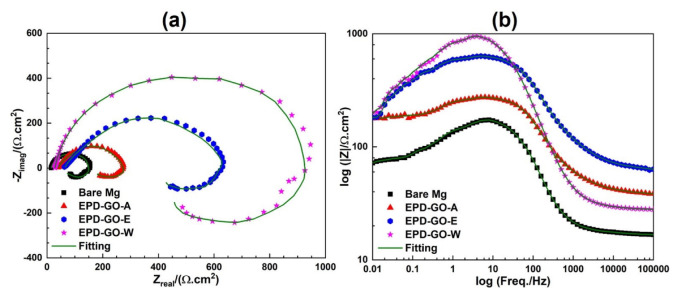
Fitted (**a**) Nyquist and (**b**) bode plot of uncoated and EPD-GO-coated Mg samples.

To corroborate corrosion inhabitation capability of EPD-GO coatings established by Tafel analysis, EIS was taken in Ringer’s lactate solution. This nondestructive technique explains how corrosion inhabitation expanded due to different EPD-GO coatings in different electrolytes [69]. Figure 8a shows the Nyquist plots of uncoated and GO-coated samples. At high- and medium-frequencies, due to the weakly bonded oxide layer on the surface, the curves display capacitive loops which indicate double-layer phenomena [70]. While at low frequencies, the inductive behavior is caused by the adsorbed species relaxation process when metal encounters Ringer’s lactate solution. Nyquist plots, exhibiting parallel capacitive loops of different sizes, indicate that all samples with different corrosion rates, obtained in Tafel analysis, used the same corrosion process [21]. Due to smooth and adherent GO coating, as seen in SEM images, it creates a torturous path for the active ion species (chloride, fluoride, and sulfite) of Ringer’s lactate solution. EPD-GO-W has a greater span of capacitive loop and avoids development of a non-adherent Mg(OH)_2_ layer; this is clearly endorsed by Tafel analysis of β_c_. 

**Figure 9:**
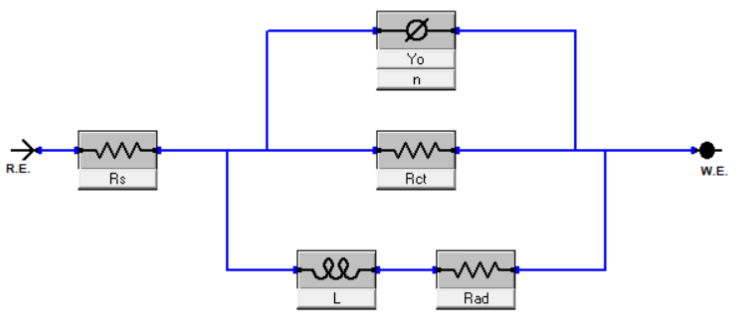
EEC model used for uncoated and EPD-GO-coated Mg samples.

To further elaborate the coatings performance, an electrical equivalent circuit (EEC) was fitted (goodness of fit ≤10^−3^). Figure 9 illustrates the EEC model and Table 2 presents all attained circuit values from fitted curves, where R_s_, R_ct_, R_ad_, L, Y_o_, and n are solution resistance, charge transfer resistance, adsorbed species resistance, inductance, admittance, and dimensionless coefficient, respectively. EPD-GO-W had ~6× better R_ct_ (873.5 Ω×cm^2^), EPD-GO-E had ~4× better R_ct_ (601.2 Ω×cm^2^), and EPD-GO-A had ~2× better R_ct_ (242.9 Ω×cm^2^) than that of bare Mg (150.3 Ω×cm^2^), which validated Tafel results. Also, R_ad_ for the EPD-GO-W coating on Mg sample was highest (299.2 Ω×cm^2^), indicating a more compacted and adherent double layer of EPD-GO coatings and electrolyte, respectively, to resist further corrosion. Due to the swelling effect of the EPD-GO coatings, as there may have been some partially reduced GO present on coatings and their hydrophilic nature, the admittance (Y_o_) values of EPD-GO coatings were lower than the values for bare Mg [30]. The equivalent inductive behavior observed in the low frequency showed GO coatings are advantageous for molding the depletion rate of the Mg alloys without impairing the characteristics of the underlying metal. A dimensionless coefficient (n) was introduced to fix deviation in admittance which suggests the surface roughness, compactness, cracks, and porosity of the deposited coatings. High and low values of n indicate better capacitive behavior and highly porous and rough surface of deposited coatings, respectively [71,72]. Here, Table 2 shows the highest value of n for EDP-GO-W, while the lowest comparable values for other EPD-GO-coated Mg samples, indicating better coverage with low roughness for EPD-GO-W coatings and well validated, with the Tafel, AFM and SEM results. 

Figure 8b presents the bode plot of uncoated and GO-coated samples at different frequency ranges. The corrosion resistance property of coatings is directly relational to the value of modulus of impedance (|Z|) at lower frequency (0.01 Hz), with higher |Z| value showing better corrosion resistance and vice versa. Here, the EPD-GO-W-coated Mg sample had the highest |Z|, about 300 Ω×cm^2^, slightly higher than that of EPD-GO-A and EPD-GO-E, which were 295 Ω×cm^2^ and 260 Ω×cm^2^, respectively. These values were all about 4× higher than the |Z| value for the uncoated Mg (80 Ω×cm^2^). This showed higher corrosion resistance behavior of GO-coated samples compared to uncoated Mg sample. It can be inferred from the corrosion tests that the EPD-GO-coated biodegradable Mg samples, especially EPD-GO-W-coated Mg samples, can provide reasonable corrosion protection during the bone restorative stage and, after relapse of EPD-GO coatings, Mg alloys will act as biodegradable. Thus, EPD-GO coatings could be able to play a major role in those implants which require protection for particular periods of time from the corrosion produced by bodily fluid. Table 3 presents the different composite coatings and results from the current study and already conducted research for better comparison. 

### 3.5. Biocompatibility

**Figure 10:**
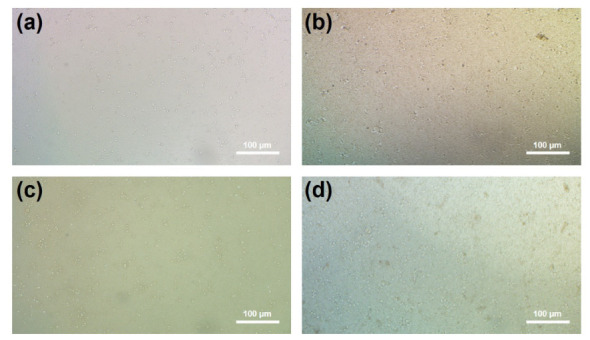
Morphologies of MC3T3-E1 osteoblast cells cultured on (**a**) bare Mg, (**b**) EPD-GO-A-, (**c**) EPD-GO-E-, and (**d**) EPD-GO-W-coated Mg samples after 3 days of incubation.

**Figure 11:**
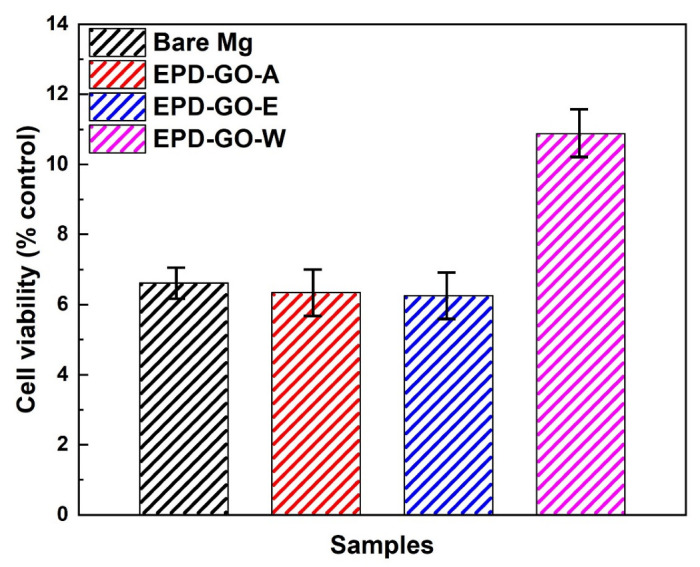
Percentage proliferation of MC3T3-E1 osteoblast cells lines cultured in media for 3 days with bare and EPD-GO-coated Mg samples.

Due to their low cost and their easy of use, MTT tests have been widely used for the quantification of cytotoxicity. The morphologies of cultured cells on bare, EPD-GO-coated Mg samples after 3 days of incubation are shown in Figure 10. Cell morphologies and viabilities of EPD-GO-A- and EPD-GO-E-coated samples were quite similar to those of the bare Mg sample, while the EPD-GO-W-coated Mg sample showed an improvement in cell viability even after 3 days of incubation. It can be deduced that the EPD-GO coatings on Mg did not change the culturing of the cells on the samples; also, good coating, as in the case of EPD-GO-W, enhance the capability of cell growth. Further, Figure S3 shows the normal media cell after 3 days of incubation. Figure 11 shows the percentage proliferation of MC3T3-E1 osteoblast cells lines cultured in media for 3 days with bare and EPD-GO-coated Mg samples. EPD-GO coatings on Mg did not produce any harmful effects on the biocompatibility of the samples, while EPD-GO-W coatings improved it by 2× as compared to bare Mg sample. These results are well validated by the previous literature, with GO coatings supporting the initial attachment, proliferation, growth, and no genotoxicity of cells [22,42,43,44]. Hence, EPD-GO coatings can be used for implants and other future applications where we need corrosion protection for a specific length of time. 

## 4. Conclusions

GO was effectively developed by improved Hummers’ method, having a thickness of ~1 nm as defined by AFM analysis. The EPD technique was used to deposit GO coatings on Mg alloy using different ultrasonicated GO suspensions of various solvents (water, ethanol and acetone). XRD of the EPD-GO-coated Mg samples confirmed the successful deposition of GO on Mg. Raman spectra verified the successful oxidation of graphite and presence of multilayer GO sheets. AFM showed the smoothness of EPD-GO-coated samples with their respective morphologies and height profiles, indicating successful deposition of GO coatings on Mg samples. SEM analysis revealed that EPD-GO coatings provide good coverage to Mg, especially EPD-GO-E- and EPD-GO-W- coated samples, while EDX spectrum confirmed the existence of GO coatings on Mg samples. Tafel analysis of EPD-GO-W coating showed 93.79% corrosion effectiveness compared to that of the bare Mg alloy in Ringer’s lactate solution, and was effectively validated by EIS analysis. This research shows that solvent has a major impact on coating deposition and corrosion properties of GO-coated samples. Deionized water, owing to its polarity, was found to be an efficient solvent, as it enabled uniform dispersion of GO thanks to the oxygen functional groups of GO. Therefore, GO coatings developed from GO water suspension resulted in better coverage and enhanced corrosion protection of AZ31B compared to coatings developed from other solvents’ suspensions. Moreover, the in vitro biocompatibility and cytotoxicity results showed EPD-GO coatings have a potential to limit the in vitro degradation rate of AZ31B Mg alloys without any harmful effects for biomedical applications.

## Figures and Tables

**Table 1:** Kinetic parameters obtained from Tafel analysis.

Sample	β_a_ (mV/decade)	β_c_ (mV/decade)	I_corr_ (μA/cm^2^)	Ε_corr_ (V)	R_p_ (Ω × cm^2^)	Avg. Corrosion Rate (mpy)	η (%)
Bare Mg	100.9	314.7	36.38 ± 0.62	−1.48	0.916	32.39	---
EPD-GO-A	91.50	163.5	4.930 ± 0.079	−1.40	5.167	4.573	86.46
EPD-GO-E	109.5	160.8	4.140 ± 0.043	−1.34	6.832	3.678	88.63
EPD-GO-W	128.4	209.4	2.260 ± 0.021	−1.21	15.29	2.004	93.79

**Table 2:** Impedance values obtained after EEC fitting.

Sample	R_s_ (Ω × cm^2^)	R_ct_ (Ω × cm^2^)	R_ad_ (Ω × cm^2^)	L (H × cm^2^)	Y_o_ (µS × s^a^/cm^2^)	n
Bare Mg	17.29	150.8	93.92	63.88	32.89	0.91 ± 0.06
EPD-GO-A	35.10	242.9	135.7	159.5	31.19	0.87 ± 0.03
EPD-GO-E	56.32	601.2	190.4	428.9	18.45	0.85 ± 0.07
EPD-GO-W	28.96	873.5	299.2	708.3	10.41	0.93 ± 0.05

**Table 3:** Different composite coatings and results from the current study and already conducted research.

Mg Alloy	Composites	Coating Technique	Electrolyte (wt.%)	Electrochemical Result	Ref.
AZ31B	GO-A, GO-E, GO-W	Electrophoretic deposition	Ringer’s lactate	~6× increase in R_ct_ for EPD-GO-W	This work
AZ31	MAO-LDHs/8-HQ@GO	Ring-opening reaction, micro-arc oxidation and hydrothermal chemical transformation	3.5% NaCl	~1.5× increase in R_ct_ even after 14 days	[73]
AZ31B	GO	Electrophoretic deposition	Ringer’s lactate	Decreased corrosion rate ~16×	[32]
AZ60	PDA/CaP/GO	Biomimetic deposition and spin-coating	SBF	~27× increase in R_ct_	[74]
AZ91	GPTMS/GO	Electroless co-deposition	3.5% NaCl	~5× increase in R_ct_	[75]
AZ91	GPTMS/GO/FAS	Electroless co-deposition	3.5% NaCl	~100% corrosion protection efficiency	[76]
AZ91	HA/GO	Biomimetic method	SBF	Improves corrosion resistance due to positive shift of polarization curves	[77]
AZ91	GO	Micro-arc oxidation process	SBF	~2.5× increase in R_ct_	[78]
AZ91D	Alumina/GO	Electrophoretic deposition	3.5% NaCl	~17.5× increase in R_ct_	[79]
ZQ71	Mg(OH)_2_/GO/HA	Electrophoretic and electrochemical deposition	PBS	~98% corrosion protection efficiency	[80]
ZK60	HA/G/GO	Hydrothermal method	PBS	Decreased corrosion rate ~28.5×	[81]
Mg-5.7Zn-0.8Ca alloy	APTES/GO	Hydrolysis process and silane agent	3.5% NaCl	~3× increase in R_ct_	[82]
Mg-4Zn- 4Sn-0.6Ca-0.5Mn	HA/chitosan/GO	Electrophoretic deposition	SBF	~2× increase in R_ct_	[16]
Mg-6.0Zn-0.5Ca alloy	Ce(Ⅳ)/GO/PVA	Spin-casting method	3.5% NaCl	~2× increase in R_ct_	[83]
Mg-3.0Zn-0.5Ca alloy	Ce/WEP/GO	Spin-coating	3.5% NaCl	Decreased corrosion rate ~550×	[84]

## Data Availability

All data is given in the manuscript.

## Supplementary Materials

The following supporting information can be downloaded at: https://www.mdpi.com/article/10.3390/nano12213745/s1, Figure S1: Schematic illustration; Figure S2: Raman spectroscopy of GO and EPD-GO-W coated sample; Figure S3: Normal media cells. References [85,86,87] were cited in Supplementary Materials.
