# Supplementary material for: Role of Solvent Used in Development of Graphene Oxide Coating on AZ31B Magnesium Alloy: Corrosion Behavior and Biocompatibility Analysis
Source: Nanomaterials (Basel). 2022 Oct 25;12(21):3745. doi: 10.3390/nano12213745 (PMC9654966; doi:10.3390/nano12213745)
Supplement: Supplementary file 1 [file nanomaterials-12-03745-s001.zip › nanomaterials-1967717-supplementary.pdf]

nanomaterials

Supplementary Materials

Role of Solvent Used in Development of Graphene Oxide Coating on AZ31B Magnesium Alloy: Corrosion Behavior and Biocompatibility Analysis

Muhammad Faheem Maqsood [1,2,3], Mohsin Ali Raza [1,*], Zaeem Ur Rehman [1], Asima Tayyeb [4], Muhammad Atif Ma-khdoom [1], Faisal Ghafoor [3], Umar Latif [1] and Muhammad Farooq Khan [3,*]

1 Institute of Metallurgy & Materials Engineering, Faculty of Chemical & Materials Engineering, University of the Punjab, Lahore 54590, Pakistan
2 Faculty of Nanotechnology and Advanced Materials Engineering, Sejong University, Seoul 05006, Korea
3 Department of Electrical Engineering, Sejong University, 209- Neungdong-ro, Gwangjin-gu, Seoul 05006, Korea
4 School of Biological Sciences, Quaid-e-Azam Campus, University of the Punjab, Lahore 54590, Pakistan

* Correspondence: mohsin.imme@pu.edu.pk (M.A.R.); mfk@sejong.ac.kr (M.F.K.)

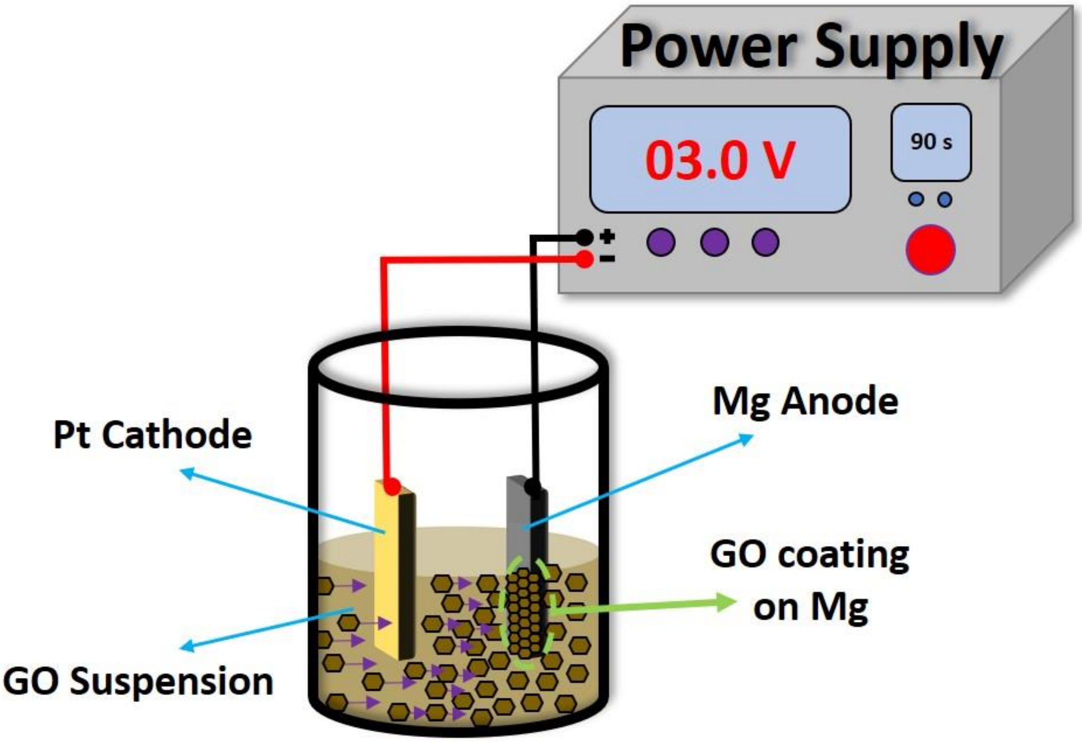

Figure S1. Schematic representation.

1. Raman spectroscopy

Raman spectroscopy is an important technique to Characterize GO. Raman spectra of as synthesized GO and EPD-GO-W sample are presented in Figure S2. Raman spectrum of GO exhibit three major peaks. G band corresponds to stretching of C-C bonds and attributes to graphitic structure. G band is present in all carbon structures [1]. On the other hand, D band shows presence of structural defects in pure graphitic structure. D band is important in GO structure as oxidation produces defects in graphite structure due to

intercalation of oxide functional groups [2,3]. While 2D band tells us about number of layers of GO. A sharp peak at 2700 cm^{-1} shows single layer while broader peak shows presence of multilayer GO [4]. Raman spectra of GO and EPD-GO-W shows D band at ca. 1360 cm^{-1} which confirms presence of disorder in graphite structure due to oxidation and G band is present at ca. 1580 cm^{-1} which is characteristic band of graphitic structure. Presence of well-defined D and G bands in EPD-GO-W sample confirmed presence of GO coating on Mg surface. I_D/I_G ratio of GO is 0.88 and EPD-GO-W is 0.99 which also confirmed presence of defects in graphite structure. Broad 2D band is present at 2700 cm^{-1} in both samples which confirmed presence of multilayer GO sheets.

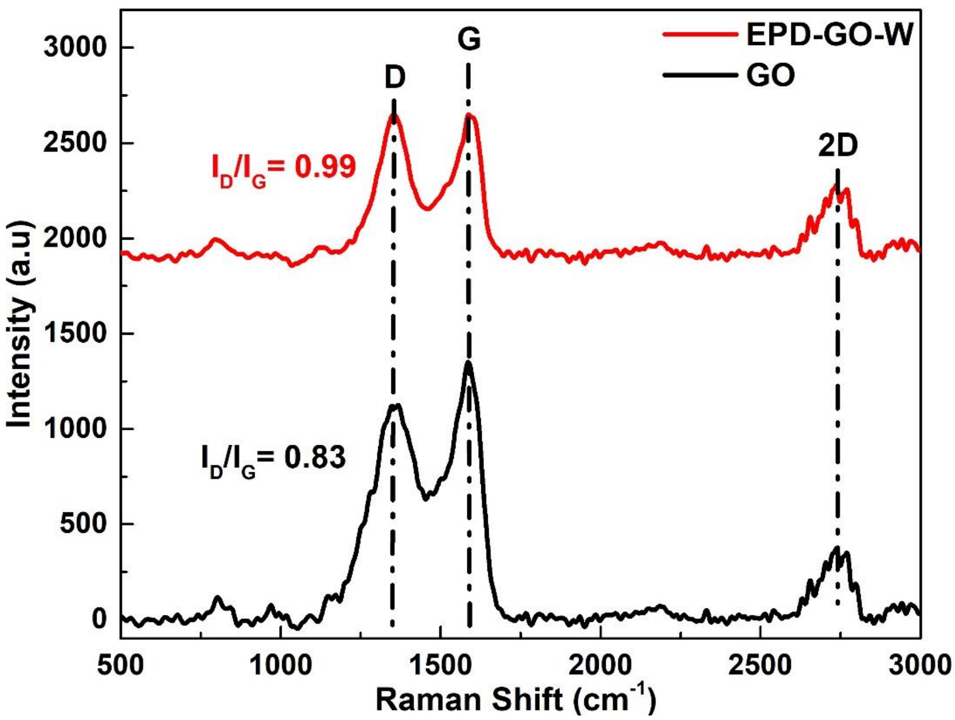

Figure S2. Raman spectra of GO and EPD-GO-W coated sample.

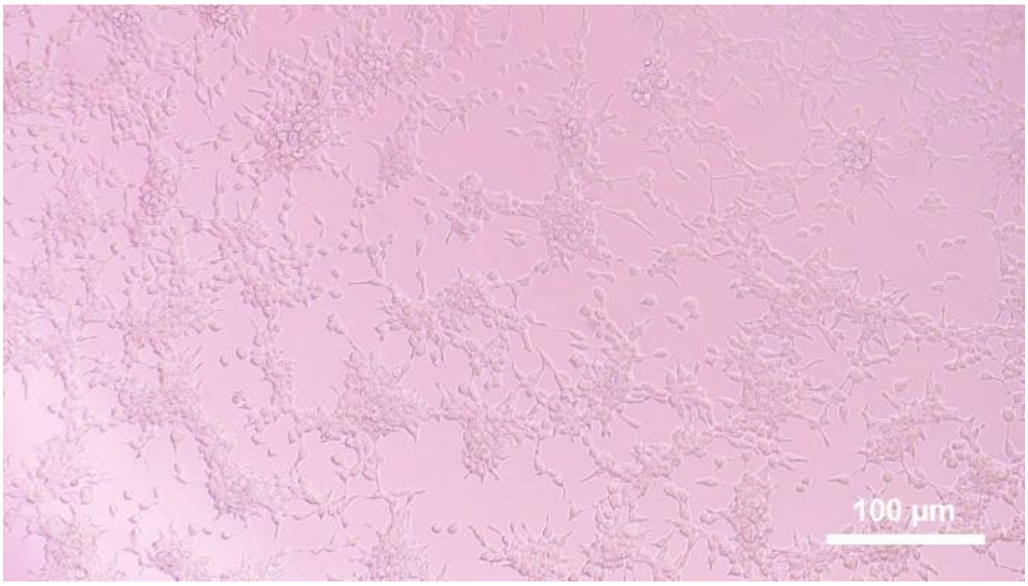

Figure S3. normal media cells.

References

1. Ferrari, A.C.; Robertson, J.; Interpretation of Raman spectra of disordered and amorphous carbon. *Physical review B*, **2000**, *61*(20): p. 14095.
2. Raza, M.A., Rehman, Z.U.; Ghauri, F.A.; Corrosion study of silane-functionalized graphene oxide coatings on copper. *Thin Solid Films*, **2018,** *663*: p. 93-99.
3. Kudin, K.N., Ozbas, B; Schniepp, H.C.; Prud'homme, R.K.; Aksay, I.A.; Car, R.; Raman spectra of graphite oxide and functionalized graphene sheets. *Nano letters*, **2008**, *8(1)*: p. 36-41.
4. Johra, F.T., Lee, J.-W.; Jung, W.-G.; Facile and safe graphene preparation on solution based platform. J. Ind. Eng. Chem. **2014**, *20(5)*: p. 2883-2887.
